# Tetra­aqua­bis­(2-{[5-(pyridin-4-yl)-1,3,4-oxadiazol-2-yl]sulfan­yl}acetato)­iron(II)

**DOI:** 10.1107/S1600536811038918

**Published:** 2011-09-30

**Authors:** Hai-Rong Wang, Guo-Ting Li

**Affiliations:** a Department of Environmental and Municipal Engineering, North China University of Water Conservancy and Electric Power, Zhengzhou, 450011, People’s Republic of China

## Abstract

In the title compound, [Fe(C_9_H_6_N_3_O_3_S)_2_(H_2_O)_4_] or [Fe(POA)_2_(H_2_O)_4_], the Fe^II^ atom is located on an inversion center and is ligated by four O atoms of coordinated water mol­ecules in the equatorial plane while two POA ligands acting as monodentate ligands occupy the axial positions through their pyridyl N atoms, completing a slightly distorted octa­hedral coordination geometry. A three-dimensional supra­molecular network is formed by multiple O—H⋯O hydrogen-bonding inter­actions between the coordinated water donors and the uncoordinated carboxyl acceptors.

## Related literature

For the synthesis of 5-(4-pyrid­yl)-1,3,4-oxadiazole-2-thione, see: Young & Wood (1955 ▶). For metal-assisted transformation of *N*-benzoyl­dithio­carbazate to 5-phenyl-1,3,4-oxadiazole-2-thiol (pot) in the presence of ethyl­enediamine, and its first-row transition-metal complexes, see: Tripathi *et al.* (2007 ▶). For Zn^II^ and Cd^II^ metal-organic polymers with the versatile building block 5-(4-pyrid­yl)-1,3,4-oxadiazole-2-thiol, see: Du *et al.* (2006 ▶).
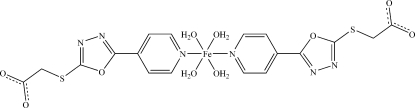


## Experimental

### 

#### Crystal data

[Fe(C_9_H_6_N_3_O_3_S)_2_(H_2_O)_4_]
*M* *_r_* = 600.37
Monoclinic, 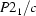

*a* = 14.365 (3) Å
*b* = 10.709 (2) Å
*c* = 7.5709 (15) Å
β = 91.45 (3)°
*V* = 1164.2 (4) Å^3^
*Z* = 2
Mo *K*α radiation
μ = 0.90 mm^−1^
*T* = 293 K
0.20 × 0.20 × 0.20 mm

#### Data collection

Siemens SMART CCD diffractometer
Absorption correction: multi-scan (*SADABS*; Sheldrick, 1996 ▶) *T*_min_ = 0.949, *T*_max_ = 1.000
12366 measured reflections
2285 independent reflections
2179 reflections with *I* > 2σ(*I*)
*R*_int_ = 0.034

#### Refinement

*R*[*F* ^2^ > 2σ(*F* ^2^)] = 0.036
*wR*(*F* ^2^) = 0.078
*S* = 1.13
2285 reflections
185 parameters
H atoms treated by a mixture of independent and constrained refinement
Δρ_max_ = 0.33 e Å^−3^
Δρ_min_ = −0.25 e Å^−3^

### 

Data collection: *SMART* (Siemens, 1996 ▶); cell refinement: *SAINT* (Siemens, 1994 ▶); data reduction: *SAINT*; program(s) used to solve structure: *SHELXS97* (Sheldrick, 2008 ▶); program(s) used to refine structure: *SHELXL97* (Sheldrick, 2008 ▶); molecular graphics: *SHELXTL* (Sheldrick, 2008 ▶); software used to prepare material for publication: *SHELXL97*.

## Supplementary Material

Crystal structure: contains datablock(s) I. DOI: 10.1107/S1600536811038918/si2366sup1.cif

Structure factors: contains datablock(s) I. DOI: 10.1107/S1600536811038918/si2366Isup2.hkl

Additional supplementary materials:  crystallographic information; 3D view; checkCIF report

## Figures and Tables

**Table 1:** Selected bond lengths (Å)

Fe1—O1	2.0605 (17)
Fe1—O2	2.1340 (18)
Fe1—N1	2.2359 (18)

**Table 2:** Hydrogen-bond geometry (Å, °)

*D*—H⋯*A*	*D*—H	H⋯*A*	*D*⋯*A*	*D*—H⋯*A*
O1—H1*A*⋯O5^i^	0.83 (3)	1.87 (3)	2.691 (3)	170 (3)
O1—H1*B*⋯O5^ii^	0.83 (3)	1.82 (4)	2.649 (2)	174 (3)
O2—H2*A*⋯O4^ii^	0.82 (3)	2.07 (3)	2.896 (3)	177 (3)
O2—H2*B*⋯O4^iii^	0.84 (4)	1.98 (4)	2.817 (3)	175 (3)

Symmetry codes: (i) 
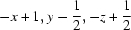
; (ii) 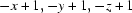; (iii) 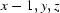.

**Fig. 1.**
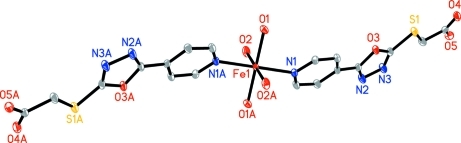
ORTEP diagram of (1) with atom numbering scheme showing coordination sphere of metal center FeII (30% probability ellipsoids for all non-hydrogen atoms). Symmetry code A: -x, -y + 1, -z.

**Fig. 2.**
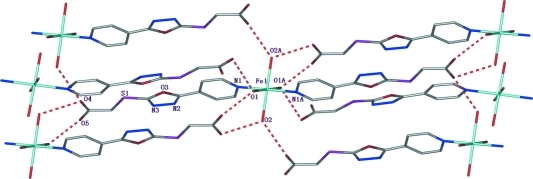
View of a section of the hydrogen-bonding system among monomers (1).

**Fig. 3.**
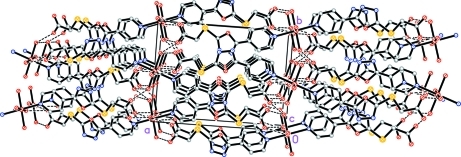
View of three-dimensional hydrogen-bonding supramolecular network.

## supplementary crystallographic information

### Comment

Recently, pyridyl-containing 1,3,4-oxadiazole-2-thione have been systematically explored as promising bridging ligands in coordination chemistry. Metal- assisted transformation of *N*-benzoyldithiocarbazate to 5-phenyl-1,3,4-oxadiazole-2-thiol (pot) in the presence of ethylenediamine, and its first row transition metal complexes were discussed by N. K. Singh and coworkers [Tripathi *et al.*, (2007)]. A report describing Zn^II^ and Cd^II^ metal-organic polymers with a versatile building block 5-(4-pyridyl) -1,3,4-oxadiazole-2-thiol was presented by Du *et al.*, (2006). We purposedly engrafted the carboxylic group into the 5-(pyridin-4-yl)-1,3,4-oxadiazol-2-ylthio backbone and synthesized the multifunctional ligands 2-(5-(pyridin-4-yl)-1,3,4-oxadiazol-2-ylthio)acetic acid (HPOA). Herein we report that the reaction of FeSO_4_.7H_2_O and sodium(I) salt of HPOA leads to a new complex [Fe(POA)_2_(H_2_O)_4_] (Fig.1).

The title compound is a mononuclear complex in which every Fe^II^ ion located at the inversion center *i* reproduces the whole molecule through the asymmetry unit consisting of one-half Fe^II^, one deprotonated POA and two water molecules. In (1) the iron(II) center is ligated by four O from water molecules in the equatorial plane, and two POA anions acting as monodentate ligands and occupy the axial positions through their pyridyl nitrogen atoms coordinating to Fe^II^, which is in an axial-elongated octahedral coordination sphere with the bond distances of Fe—O and Fe—N ranging from 2.0605 (17) to 2.2359 (18) Å (Table 1).

In (1) the uncoordinated carboxyl groups as typical hydrogen-bonding acceptors are authentically interesting in the construction of an intricate three-dimensional supramolecular network. Clearly, further aggregation of the monomers (1) is directed by the multiple hydrogen-bonding between the coordinated water donors and the uncoordinated carboxyl acceptors. Fig. 2 shows the complicated hydrogen-bonding system among monomers (1): each coordination water molecule in one monomer forms two O—H···O hydrogen bonds (Table 2) with carboxyl groups to bridge two monomers, while every carboxyl group of POA in the monomer acts as a three-connected hydrogen-bonding acceptor and adopts two different hydrogen-bonding models (bridging and chelating modes) to links with three monomers. Consequently, every monomer acts as a novel six-connected supramolecular synthon to connect with six adjacent monomers. In this way monomers (1) are arrayed to create a three-dimensional supramolecular architecture as shown in Fig. 3.

### Experimental

5-(4-pyridyl)-1,3,4-oxadiazole-2-thione was synthesized according to the reported method (Young & Wood, 1955). The sodium(I) salt of the ligand 2-(5-(pyridin-4-yl)-1,3,4-oxadiazol-2-ylthio)acetic acid (HPOA) was synthesized as the following process. To a solution of sodium hydroxide (1.60 g, 40 mmol) and 95% alcohol (50 ml) was added 5-pyridyl-2-mercapto-1,3,4-oxadiazole (3.58 g, 20 mmol) and the resulting mixture was refluxed for half an hour. And then a solution of chloroactic acid (1.89 g, 20 mmol) and 95% alcohol (70 ml) was dropwise added to the mixture with continuous refluxing for 3 h. Pale yellow precipitate was filtered. After recrystallized from alcohol/water (2:1), the obtained pure product was 2.76 g. Yield: 51%. Selected IR (cm^-1^, KBr pellet): 3489(w), 1598(*s*), 1464(*m*), 1402(*s*), 1220(*m*), 1190(*m*), 1084(*m*), 909(*m*), 835(*m*), 704(w), 519(*m*).

The title compound (1), was prepared according to the following process. A mixture of NaPOA (51.8 mg, 0.2 mmol), FeSO_4_.7H_2_O (27.8 mg, 0.1 mmol) and deionized water (20 ml) was stirred for 30 minutes and then filtered. The filtrate was allowed to evaporate at room temperature for three days, and yellow crystals were obtain in 36% yield. Selected IR (cm^-1^, KBr pellet): 3416(*m*), 3194(*m*), 1618(*s*), 1545(*s*), 1495(w), 1450(*s*), 1423(w), 1379(*s*), 1226(*m*), 1198(*m*), 1087(w), 1063(w), 1003(w), 871(w), 840(w), 799(w), 743(w), 707(*s*), 586(w), 522(w).

### Refinement

The H atoms of water molecules were located from difference Fourier maps, and their positional and isotropic displacement parameters were refined, while the other hydrogen atoms were assigned with common isotropic displacement factors [*U*_iso_(H) = 1.2 times *U*_eq_(C)] and included in the final refinement by using geometrical restraints.

### Crystal data

**Table:**

[Fe(C_9_H_6_N_3_O_3_S)_2_(H_2_O)_4_]	*F*(000) = 616
*M**_r_* = 600.37	*D*_x_ = 1.713 Mg m^−^^3^
Monoclinic, *P*2_1_/*c*	Mo *K*α radiation, λ = 0.71073 Å
Hall symbol: -P 2ybc	Cell parameters from 3319 reflections
*a* = 14.365 (3) Å	θ = 2.4–30.9°
*b* = 10.709 (2) Å	µ = 0.90 mm^−^^1^
*c* = 7.5709 (15) Å	*T* = 293 K
β = 91.45 (3)°	Prism, yellow
*V* = 1164.2 (4) Å^3^	0.20 × 0.20 × 0.20 mm
*Z* = 2	

### Data collection

**Table:**

Siemens SMART CCD diffractometer	2285 independent reflections
Radiation source: fine-focus sealed tube	2179 reflections with *I* > 2σ(*I*)
graphite	*R*_int_ = 0.034
ω scan	θ_max_ = 26.0°, θ_min_ = 2.4°
Absorption correction: multi-scan (*SADABS*; Sheldrick, 1996)	*h* = −17→17
*T*_min_ = 0.949, *T*_max_ = 1.000	*k* = −13→13
12366 measured reflections	*l* = −9→9

### Refinement

**Table:**

Refinement on *F*^2^	Primary atom site location: structure-invariant direct methods
Least-squares matrix: full	Secondary atom site location: difference Fourier map
*R*[*F*^2^ > 2σ(*F*^2^)] = 0.036	Hydrogen site location: inferred from neighbouring sites
*wR*(*F*^2^) = 0.078	H atoms treated by a mixture of independent and constrained refinement
*S* = 1.13	*w* = 1/[σ^2^(*F*_o_^2^) + (0.0327*P*)^2^ + 0.5694*P*] where *P* = (*F*_o_^2^ + 2*F*_c_^2^)/3
2285 reflections	(Δ/σ)_max_ < 0.001
185 parameters	Δρ_max_ = 0.33 e Å^−^^3^
0 restraints	Δρ_min_ = −0.25 e Å^−^^3^

### Special details

**Table:**

Geometry. All e.s.d.'s (except the e.s.d. in the dihedral angle between two l.s. planes) are estimated using the full covariance matrix. The cell e.s.d.'s are taken into account individually in the estimation of e.s.d.'s in distances, angles and torsion angles; correlations between e.s.d.'s in cell parameters are only used when they are defined by crystal symmetry. An approximate (isotropic) treatment of cell e.s.d.'s is used for estimating e.s.d.'s involving l.s. planes.
Refinement. Refinement of *F*^2^ against ALL reflections. The weighted *R*-factor *wR* and goodness of fit *S* are based on *F*^2^, conventional *R*-factors *R* are based on *F*, with *F* set to zero for negative *F*^2^. The threshold expression of *F*^2^ > σ(*F*^2^) is used only for calculating *R*-factors(gt) *etc*. and is not relevant to the choice of reflections for refinement. *R*-factors based on *F*^2^ are statistically about twice as large as those based on *F*, and *R*- factors based on ALL data will be even larger.

### Fractional atomic coordinates and isotropic or equivalent isotropic displacement parameters (Å^2^)

**Table:**

	*x*	*y*	*z*	*U*_iso_*/*U*_eq_	
Fe1	0.0000	0.5000	0.0000	0.02246 (14)	
S1	0.65085 (4)	0.54011 (6)	0.38712 (8)	0.03380 (17)	
O1	0.03673 (11)	0.31754 (16)	0.0529 (3)	0.0319 (4)	
O2	−0.01063 (14)	0.52700 (19)	0.2780 (2)	0.0397 (5)	
O3	0.48086 (10)	0.57082 (15)	0.2519 (2)	0.0302 (4)	
O4	0.84192 (11)	0.52207 (17)	0.5162 (2)	0.0407 (4)	
O5	0.84840 (11)	0.70106 (16)	0.6691 (2)	0.0397 (4)	
N1	0.14562 (12)	0.57012 (18)	0.0402 (2)	0.0272 (4)	
N2	0.44244 (13)	0.76466 (19)	0.3170 (3)	0.0381 (5)	
N3	0.53408 (13)	0.74249 (19)	0.3821 (3)	0.0378 (5)	
C1	0.21910 (15)	0.4960 (2)	0.0133 (3)	0.0281 (5)	
H1	0.2093	0.4211	−0.0516	0.034*	
C2	0.30836 (15)	0.5226 (2)	0.0746 (3)	0.0298 (5)	
H2	0.3582	0.4665	0.0544	0.036*	
C3	0.16251 (15)	0.6786 (2)	0.1237 (3)	0.0329 (5)	
H3	0.1119	0.7341	0.1398	0.039*	
C4	0.24920 (15)	0.7138 (2)	0.1873 (3)	0.0331 (5)	
H4	0.2580	0.7920	0.2446	0.040*	
C5	0.32349 (14)	0.6327 (2)	0.1658 (3)	0.0259 (5)	
C6	0.41495 (14)	0.6630 (2)	0.2443 (3)	0.0277 (5)	
C7	0.55192 (14)	0.6288 (2)	0.3411 (3)	0.0288 (5)	
C8	0.70959 (16)	0.6582 (2)	0.5169 (3)	0.0367 (6)	
H8A	0.7116	0.7366	0.4478	0.044*	
H8B	0.6736	0.6747	0.6243	0.044*	
C9	0.80811 (15)	0.6208 (2)	0.5712 (3)	0.0312 (5)	
H2A	0.037 (2)	0.516 (3)	0.338 (4)	0.053 (9)*	
H2B	−0.056 (3)	0.522 (3)	0.346 (5)	0.068 (11)*	
H1A	0.067 (2)	0.281 (3)	−0.024 (4)	0.051 (9)*	
H1B	0.070 (2)	0.312 (3)	0.145 (5)	0.066 (11)*	

### Atomic displacement parameters (Å^2^)

**Table:**

	*U*^11^	*U*^22^	*U*^33^	*U*^12^	*U*^13^	*U*^23^
Fe1	0.0168 (2)	0.0266 (2)	0.0237 (2)	0.00109 (17)	−0.00395 (17)	0.00006 (17)
S1	0.0217 (3)	0.0386 (3)	0.0406 (4)	0.0032 (2)	−0.0081 (2)	−0.0074 (3)
O1	0.0271 (9)	0.0336 (9)	0.0345 (10)	0.0056 (7)	−0.0080 (8)	0.0003 (8)
O2	0.0267 (9)	0.0674 (13)	0.0250 (9)	0.0050 (9)	−0.0024 (8)	−0.0034 (8)
O3	0.0180 (8)	0.0341 (9)	0.0380 (9)	−0.0009 (6)	−0.0067 (7)	−0.0049 (7)
O4	0.0249 (9)	0.0502 (11)	0.0468 (11)	0.0041 (8)	−0.0009 (8)	0.0008 (9)
O5	0.0322 (9)	0.0462 (10)	0.0399 (10)	−0.0095 (8)	−0.0174 (8)	0.0072 (8)
N1	0.0174 (9)	0.0328 (11)	0.0311 (10)	−0.0016 (8)	−0.0037 (8)	0.0004 (8)
N2	0.0216 (10)	0.0379 (12)	0.0539 (13)	0.0011 (8)	−0.0125 (9)	−0.0074 (10)
N3	0.0223 (10)	0.0368 (12)	0.0536 (13)	0.0004 (8)	−0.0136 (9)	−0.0090 (10)
C1	0.0229 (11)	0.0314 (12)	0.0298 (11)	−0.0027 (9)	−0.0001 (9)	−0.0026 (9)
C2	0.0191 (11)	0.0351 (13)	0.0352 (12)	0.0029 (9)	−0.0010 (9)	−0.0024 (10)
C3	0.0209 (11)	0.0311 (12)	0.0464 (14)	0.0024 (9)	−0.0052 (10)	−0.0042 (11)
C4	0.0267 (12)	0.0297 (12)	0.0425 (14)	−0.0005 (10)	−0.0043 (10)	−0.0050 (10)
C5	0.0176 (10)	0.0337 (12)	0.0262 (11)	−0.0044 (9)	−0.0036 (9)	0.0042 (9)
C6	0.0177 (10)	0.0338 (13)	0.0315 (12)	0.0011 (9)	−0.0012 (9)	0.0009 (10)
C7	0.0184 (10)	0.0376 (13)	0.0302 (12)	−0.0044 (9)	−0.0041 (9)	−0.0014 (10)
C8	0.0270 (12)	0.0391 (14)	0.0433 (14)	−0.0001 (10)	−0.0134 (11)	−0.0039 (11)
C9	0.0230 (11)	0.0401 (14)	0.0303 (12)	−0.0037 (10)	−0.0028 (9)	0.0108 (11)

### Geometric parameters (Å, °)

**Table:**

Fe1—O1^i^	2.0605 (17)	N1—C3	1.341 (3)
Fe1—O1	2.0605 (17)	N2—C6	1.278 (3)
Fe1—O2	2.1340 (18)	N2—N3	1.414 (3)
Fe1—O2^i^	2.1340 (18)	N3—C7	1.284 (3)
Fe1—N1^i^	2.2359 (18)	C1—C2	1.382 (3)
Fe1—N1	2.2359 (18)	C1—H1	0.9500
S1—C7	1.737 (2)	C2—C5	1.381 (3)
S1—C8	1.799 (2)	C2—H2	0.9500
O1—H1A	0.83 (3)	C3—C4	1.377 (3)
O1—H1B	0.83 (3)	C3—H3	0.9500
O2—H2A	0.82 (3)	C4—C5	1.389 (3)
O2—H2B	0.84 (4)	C4—H4	0.9500
O3—C7	1.360 (2)	C5—C6	1.464 (3)
O3—C6	1.368 (3)	C8—C9	1.517 (3)
O4—C9	1.240 (3)	C8—H8A	0.9900
O5—C9	1.265 (3)	C8—H8B	0.9900
N1—C1	1.340 (3)		
			
O1^i^—Fe1—O1	180.0	N1—C1—H1	118.2
O1^i^—Fe1—O2	92.22 (8)	C2—C1—H1	118.2
O1—Fe1—O2	87.78 (8)	C5—C2—C1	118.5 (2)
O1^i^—Fe1—O2^i^	87.78 (8)	C5—C2—H2	120.8
O1—Fe1—O2^i^	92.22 (8)	C1—C2—H2	120.8
O2—Fe1—O2^i^	180.00 (11)	N1—C3—C4	123.6 (2)
O1^i^—Fe1—N1^i^	93.33 (7)	N1—C3—H3	118.2
O1—Fe1—N1^i^	86.67 (7)	C4—C3—H3	118.2
O2—Fe1—N1^i^	95.14 (8)	C3—C4—C5	118.6 (2)
O2^i^—Fe1—N1^i^	84.86 (8)	C3—C4—H4	120.7
O1^i^—Fe1—N1	86.67 (7)	C5—C4—H4	120.7
O1—Fe1—N1	93.33 (7)	C2—C5—C4	118.74 (19)
O2—Fe1—N1	84.86 (8)	C2—C5—C6	121.4 (2)
O2^i^—Fe1—N1	95.14 (8)	C4—C5—C6	119.9 (2)
N1^i^—Fe1—N1	180.0	N2—C6—O3	112.99 (18)
C7—S1—C8	95.46 (11)	N2—C6—C5	128.9 (2)
Fe1—O1—H1A	116 (2)	O3—C6—C5	118.03 (19)
Fe1—O1—H1B	112 (2)	N3—C7—O3	113.64 (19)
H1A—O1—H1B	105 (3)	N3—C7—S1	129.59 (17)
Fe1—O2—H2A	116 (2)	O3—C7—S1	116.75 (17)
Fe1—O2—H2B	132 (2)	C9—C8—S1	112.55 (17)
H2A—O2—H2B	108 (3)	C9—C8—H8A	109.1
C7—O3—C6	101.62 (17)	S1—C8—H8A	109.1
C1—N1—C3	116.77 (19)	C9—C8—H8B	109.1
C1—N1—Fe1	121.21 (15)	S1—C8—H8B	109.1
C3—N1—Fe1	120.82 (15)	H8A—C8—H8B	107.8
C6—N2—N3	106.39 (19)	O4—C9—O5	126.8 (2)
C7—N3—N2	105.35 (18)	O4—C9—C8	120.3 (2)
N1—C1—C2	123.7 (2)	O5—C9—C8	112.9 (2)
			
O1^i^—Fe1—N1—C1	−152.98 (18)	C3—C4—C5—C2	2.9 (3)
O1—Fe1—N1—C1	27.02 (18)	C3—C4—C5—C6	−175.1 (2)
O2—Fe1—N1—C1	114.48 (18)	N3—N2—C6—O3	−0.5 (3)
O2^i^—Fe1—N1—C1	−65.52 (18)	N3—N2—C6—C5	175.9 (2)
N1^i^—Fe1—N1—C1	7(44)	C7—O3—C6—N2	0.8 (3)
O1^i^—Fe1—N1—C3	39.94 (18)	C7—O3—C6—C5	−176.01 (19)
O1—Fe1—N1—C3	−140.06 (18)	C2—C5—C6—N2	172.6 (2)
O2—Fe1—N1—C3	−52.59 (18)	C4—C5—C6—N2	−9.4 (4)
O2^i^—Fe1—N1—C3	127.41 (18)	C2—C5—C6—O3	−11.1 (3)
N1^i^—Fe1—N1—C3	−161 (44)	C4—C5—C6—O3	166.9 (2)
C6—N2—N3—C7	0.0 (3)	N2—N3—C7—O3	0.6 (3)
C3—N1—C1—C2	3.6 (3)	N2—N3—C7—S1	−177.87 (18)
Fe1—N1—C1—C2	−164.01 (18)	C6—O3—C7—N3	−0.8 (3)
N1—C1—C2—C5	−1.3 (4)	C6—O3—C7—S1	177.81 (15)
C1—N1—C3—C4	−2.6 (3)	C8—S1—C7—N3	3.4 (3)
Fe1—N1—C3—C4	165.05 (19)	C8—S1—C7—O3	−174.96 (18)
N1—C3—C4—C5	−0.6 (4)	C7—S1—C8—C9	−174.28 (18)
C1—C2—C5—C4	−2.0 (3)	S1—C8—C9—O4	4.8 (3)
C1—C2—C5—C6	176.0 (2)	S1—C8—C9—O5	−177.06 (17)

Symmetry codes: (i) −*x*, −*y*+1, −*z*.

### Hydrogen-bond geometry (Å, °)

**Table:**

*D*—H···*A*	*D*—H	H···*A*	*D*···*A*	*D*—H···*A*
O1—H1A···O5^ii^	0.83 (3)	1.87 (3)	2.691 (3)	170 (3)
O1—H1B···O5^iii^	0.83 (3)	1.82 (4)	2.649 (2)	174 (3)
O2—H2A···O4^iii^	0.82 (3)	2.07 (3)	2.896 (3)	177 (3)
O2—H2B···O4^iv^	0.84 (4)	1.98 (4)	2.817 (3)	175 (3)

Symmetry codes: (ii) −*x*+1, *y*−1/2, −*z*+1/2; (iii) −*x*+1, −*y*+1, −*z*+1; (iv) *x*−1, *y*, *z*.
